# Influences on Adaptive Planning to Reduce Flood Risks among Parishes in South Louisiana

**DOI:** 10.3390/w8020057

**Published:** 2016-02-06

**Authors:** Mary Paille, Margaret Reams, Jennifer Argote, Nina S.-N. Lam, Ryan Kirby

**Affiliations:** Department of Environmental Sciences, School of the Coast and the Environment, Louisiana State University, 1271 Energy Coast and Environment Building, Louisiana State University, Baton Rouge, LA 70803, USA

**Keywords:** planning, resilience, adaptive governance, community rating system, NFIP

## Abstract

Residents of south Louisiana face a range of increasing, climate-related flood exposure risks that could be reduced through local floodplain management and hazard mitigation planning. A major incentive for community planning to reduce exposure to flood risks is offered by the Community Rating System (CRS) of the National Flood Insurance Program (NFIP). The NFIP encourages local collective action by offering reduced flood insurance premiums for individual policy holders of communities where suggested risk-reducing measures have been implemented. This preliminary analysis examines the extent to which parishes (counties) in southern Louisiana have implemented the suggested policy actions and identifies key factors that account for variation in the implementation of the measures. More measures implemented results in higher CRS scores. Potential influences on scores include socioeconomic attributes of residents, government capacity, average elevation and past flood events. The results of multiple regression analysis indicate that higher CRS scores are associated most closely with higher median housing values. Furthermore, higher scores are found in parishes with more local municipalities that participate in the CRS program. The number of floods in the last five years and the revenue base of the parish does not appear to influence CRS scores. The results shed light on the conditions under which local adaptive planning to mitigate increasing flood risks is more likely to be implemented and offer insights for program administrators, researchers and community stakeholders.

## 1. Introduction

Since Hurricanes Katrina and Rita of 2005, risk awareness has grown among stakeholders of coastal Louisiana communities facing increasing flood risks from sea-level rise, intense storms and land subsidence. Like many other coastal regions, population growth along the Louisiana coast combined with limited land use planning has exacerbated these risks. For example, by the end of the 21st century, annual flood costs in the United States could increase from $2 billion to $7–$19 billion because of climate change, urbanization and urban emissions [1]. The National Flood Insurance Program (NFIP), designed to provide affordable insurance to property owners in flood-prone areas, is running a $25 billion deficit in the wake of recent catastrophic storms [2]. Efforts by the U.S. Congress in 2012 to increase policyholders’ premiums to more accurately cover the costs of property insurance in high risk regions were met with intense opposition from coastal stakeholders [3,4]. Given the inherent political and scientific challenges involved in setting and collecting higher premium rates for NFIP policyholders, the role to be played by local communities in formulating and implementing proactive planning to reduce overall exposure risks becomes even more important.

The Community Rating System (CRS) of the NFIP provides incentives to local communities to enact collective measures to mitigate flood risks. This analysis builds on the earlier work of several studies that examined contextual factors that may explain variation in CRS participation and helped shed light on the conditions under which local collective action may be more likely. This is especially relevant for researchers and stakeholders of Louisiana, where no previous study has examined CRS participation and given the historical ambivalence among counties and local communities concerning planning and land use management efforts [5]. Although proactive planning could help Louisiana communities increase resiliency to large-scale disturbances, enacting such land use plans requires technical information, economic resources and political will. As a result, collective actions may be more difficult to formulate and implement in some communities.

The objectives of this study are to examine the CRS participation rates and performance of parishes (counties) in south Louisiana and to identify key factors associated with greater implementation of the CRS flood risk-reducing measures.

### 1.1. The Community Rating System

The CRS is a voluntary incentive program designed to encourage communities to implement structural and non-structural flood risk-reduction measures beyond minimum NFIP requirements. Participating communities are evaluated and given a score based on the number of planning milestones they have met. The CRS scores reflect a range of activities, including implementation of land-use controls, such as preservation of floodplain as open space, regulation of development in flood-risk areas and watersheds and development of a comprehensive floodplain management plan. These measures result in a discounted flood insurance rate for National Flood Insurance Program (NFIP) policyholders in that community. NFIP discounts flood insurance rates based on a point system that ranges from 5% to 45%, increasing in 5% increments, corresponding to the score, or total number of points received [6,7].

CRS communities vary in size and may include local municipalities and parishes. Each jurisdiction within a parish has the opportunity to participate in the CRS program and is not considered part of a county-wide CRS program. In other words, if decision makers of an incorporated municipality want CRS program discounts, they must enact their own separate CRS program, distinct from the county program. Thus, the county-level CRS programs cover residents and communities within the unincorporated areas of the county.

The CRS program seeks to further three broad goals: to reduce and avoid flood damage to insurable property; to strengthen and support insurance aspects of the NFIP; and to foster comprehensive floodplain management. Following reorganization in 2013, the program focuses on six core flood-loss reduction areas: reduction of liabilities to the NFIP fund; improvement of disaster resiliency and sustainability of communities; integration of a “Whole Community” approach to address emergency management; promotion of natural and beneficial functions of floodplains; increased understanding of risk; and adoption and enforcement of disaster-resistant building codes [6]. The CRS encourages 19 activities or measures, organized into four categories: public information, mapping and regulations, flood damage reduction (structural and non-structural) and flood preparedness. Communities can also request that FEMA review other flood risk-reduction measures not listed in the program for additional CRS points.

Table 1 summarizes the types of planning and policy activities that are encouraged through the CRS program. The table shows the various activities under which communities can earn points through the CRS program, grouped into four categories (Series 300, 400, 500, 600). Each activity has a maximum number of points obtainable; however, most communities do not obtain the maximum amount of points. An average for all CRS communities in the program and an average for Louisiana communities are also included as a reference.

#### 1.1.1. Public Information Activities (300 Series)

Measures under this category include those that advise people about the flood hazard, encourage the purchase of flood insurance and provide information about ways to reduce flood damage. These activities also generate data needed by insurance agents for accurate flood insurance rating. They generally serve all members of the community.

#### 1.1.2. Mapping and Regulations (400 Series)

This series credits programs that provide increased protection to new development. These activities include mapping areas not shown on the Flood Insurance Rate Maps (FIRMs), preserving open space, protecting natural floodplain functions, enforcing higher regulatory standards and managing stormwater. The credit is increased for growing communities.

#### 1.1.3. Flood Damage Reduction Activities (500 Series)

These measures attempt to protect existing development, which is considered to be at risk within the participating jurisdiction. Credit is provided for a comprehensive floodplain management plan, relocating or retrofitting flood-prone structures and maintaining drainage systems.

#### 1.1.4. Warning and Response (600 Series)

This series provides credit for measures that protect life and property during a flood, through flood warning and response programs. There is credit for the maintenance of levees and dams and also for programs that prepare for their potential failure.

Community class rankings in the CRS range from 1 to 10. A Class 1 community can receive the highest insurance rate discount of 45%. A Class 9 community can receive a 5% discount. A Class 10 community either has failed to receive a minimum number of points or has become inactive in the program and does not receive a discount. In order for a community to become a member of the CRS program, it must be in good standing with NFIP regulations (has adopted and enforced NFIP floodplain management regulations that conform to NFIP standards) and appoint a CRS coordinator to handle all application work. Further, the CRS requires that communities actually implement these plans and monitor activities annually as a condition for renewal. Each year, communities must re-certify under the CRS program to ensure that the community is still performing the tasks for which it has received CRS points. Furthermore, a new CRS class will not be enacted until the next point tier is reached. Therefore, a community with 1000 points will have the same CRS class of 8 as a community with 1498 points. CRS class changes occur in May and October of each year. If the community does not renew each year, its residents will lose any NFIP rate discounts [8,9]. It is noteworthy that residents living in the more flood-prone Special Flood Hazard Area (SFHA) are required to have flood insurance, and most purchase policies through the NFIP.

Table 2 displays the NFIP insurance premium reductions associated with the total CRS points and the number of Louisiana parishes in each of the rate-reduction categories.

As of October 2015, the CRS program had 1368 participating communities in the United States, or approximately 5% of the total NFIP communities present. Roseville, California, is the only Class 1 ranked community in the United States [10]. Louisiana currently has 46 communities participating in the program. Of those 46, 16 are parishes and 30 are municipalities of varying size and population [11].

### 1.2. CRS Activities and Community Resilience

Historically, Louisiana communities have been slow to adopt planning measures [12], despite the potential benefits in terms of reducing exposure to flood risks. As a largely rural state, many parishes lack the resources to implement and maintain parish-wide measures, such as open-space preservation or floodplain management. Furthermore, since stakeholders of many smaller and more rural communities do not feel the pressure to implement growth management strategies, they may not recognize the benefits or relevance of planning in terms of disaster prevention and/or flood reduction [13,14]. However, smarter growth strategies and other land use planning measures may lessen the vulnerability (and increase the resiliency) of a community [15,16]. Common examples of smarter growth strategies include growth restrictions in flood-prone areas and tighter building codes and regulations [17]. However, as pressure for more development and housing grows, pressure to develop in floodplains increases, and therefore, more individual properties are exposed to risk [18–20].

In 2007, the United States Federal Emergency Management Agency (FEMA) ranked Florida, California, Texas, Louisiana and New Jersey respectively as highest risk for flooding based on a composite risk score derived from floodplain area, per capita housing and number of housing. Researchers found that “non-structural” methods, such as those measured by the CRS rating, were more than twice as effective as “structural” measures, such as dams, at reducing the level of damage from flooding [11,18]. Furthermore, while structural measures directly reduce flooding risk to property and communities, they can encourage development in flood-prone areas that are now protected by these measures [2]. Therefore, the types of measures encouraged by the CRS program, such as open space preservation, stormwater management and flood information disclosure, address an obvious need.

In England, the Netherlands and Germany, strong flood mapping tools drive planning decisions, as flood management efforts focus increasingly on non-structural methods. However, these tools still run the risk of remaining just that: tools. These programs still see resistance between central and local governments, individuals and professional planners [21–23]. Furthermore, even with increasing flooding events, research suggests individuals and organizations tend to minimize flooding events as recent as 10 years prior and see those events as isolated incidents, which are unlikely to occur again [18].

The CRS program with its incentives to individual NFIP policyholders, prescription of collective risk-reducing measures and annual evaluation of participating jurisdictions is an important resource for local decision makers seeking to reduce flood exposure risks. Previous research has shown that the CRS program does in fact promote discount-seeking activities [24,25]. The CRS program also introduces more interactions between local policy makers and citizens through the creation of specific risk assessments, information sharing and other educational outreach activities. Related research also shows that mitigation measures can be affected at the individual level through public information activities and hazard information disclosure, a large part of the CRS-creditable activities [26]. As such, the program may enhance public understanding of flood risks. According to Jennifer Gerbasi, the CRS coordinator for Terrebonne parish who was interviewed for this study, the CRS program promotes greater levels of trust in local officials for residents and encourages community-based decision making to reduce flood exposure risks [27].

Participation in the CRS program may encourage and support several key attributes of more resilient communities, as identified by resilience theorists. For example, Adger [28] and colleagues identified as a key attribute of resilience the ability to withstand repeated disturbances, like large-scale storms and floods, while still maintaining “essential structures, processes and feedbacks” within the system. The constituent members of resilient communities are able to “self-organize” to carry out essential functions in the aftermath of disturbances and are able to learn from their experiences and to adapt to reduce future exposure risks [28–32]. Researchers have observed that the process of recovering from major disturbances presents opportunities for expanded learning environments with greater stakeholder input into collective decisions and consideration of data from multiple sources to gain a more holistic understanding of the risks [33–35]. As a result, the public may become more involved, aware and informed of potential risks, and the political will to take collective action may increase.

Despite the opportunity to learn and adapt following large disturbances, lack of available information on flooding, inundation, land use and growth patterns can present challenges for community stakeholders to participate in informed decision making and for decision makers to formulate and implement proactive disaster management planning [36]. Furthermore, some communities in Louisiana have historically avoided land use planning, as a result of strong private property rights. Prior to Hurricane Katrina in 2005, Louisiana was among the states least likely to limit private property rights regarding planning and development and had not updated state-wide planning mandates put into place in 1927 [5,11]. While Hurricane Katrina spurred planning initiatives with the Louisiana Speaks program, Louisiana still lacks large-scale or state-wide planning efforts, with the Coastal Master Plan as the largest current planning effort.

Thus, the CRS program has an important role to play in Louisiana as community stakeholders work to reduce flood exposure risks. What factors may explain variation in parish-level measures for floodplain management and hazard mitigation evaluated under the CRS program? We turn to recent related research that considers the preconditions and attributes of more resilient communities, and the specific influences on CRS participation in particular, to select variables to include in our analysis.

### 1.3. Factors Associated with Disaster Resilience

In recent years, researchers have attempted to identify the most suitable indicators to assess disaster resilience. For example, in 2010, Cutter and colleagues [30] introduced the Baseline Resilience Indicator for Communities (BRIC), which is an aggregation of five sub-indexes measuring socioeconomic, institutional, infrastructural and other community capacities and attributes. Furthermore, in 2010, Sherrieb and others [8] reduced 88 variables to a smaller group of 17 variables representing two components, including social capital and economic development, as indicators of resilience. In 2015, we applied the Resilience Inference Measurement (RIM) model to measure resilience in the 52 counties of the U.S. Gulf Coast region and identified key predictors for the ability of a county to withstand exposure and damages from storms and still maintain or increase in population over time [37]. Specific factors associated with greater resilience were found to be higher elevation and greater socioeconomic resources.

Several studies have examined influences on community and household-level disaster planning. First, experience with recent floods has been found to be associated with greater community interest in and acceptance of collective planning efforts [38]. Regarding household-level measures to mitigate damages associated with floods, a survey of Tennessee residents found that individuals living in communities that experienced floods within the last year were more likely to purchase flood insurance policies [39]. The heightened awareness of flood impacts appears to have influenced residents to take action to protect their property from future floods. It is noteworthy that since 1973, the NFIP has required all properties located within the Special Flood Hazard Area to have flood insurance. However, in the Tennessee study, four years after the flood event, the number of household policies purchased through the NFIP declined, indicating a possible short-term bias in residents’ risk perceptions. Similarly, Browne and Hoyt [40] found that insurance purchases are highly correlated to the level of flood losses experienced during the previous year. Others showed that proximity to flood hazards increased the likelihood that residents will purchase flood insurance [41].

Other potential influences on parish-level adaptive planning in general are the capacities and resources of the parish government. Since planning occurs at the sub-federal and sub-state level [42,43], the resources available to local policy makers may help shape planning activities and outcomes. County and local governments play an important role both in educating residents about flood risks and developing proactive disaster planning to mitigate future damages [44]. Larger county governments with more resources and staff may be better able to implement adaptive planning measures. Furthermore, stakeholders of wealthier communities have more assets to protect and inherently have a greater stake in how those assets are protected and, thus, may be likely to support more planning [10].

Finally, recent studies examining specific influences on CRS participation and implementation of risk-reducing measures point to the importance of hydrological conditions, the socioeconomic attributes of residents and government capacity. Our study builds most directly on the work of Landry and Li (2012) in which they examined the CRS participation of 100 counties in North Carolina from 1991 to 1996 [45]. They tested the influence of factors, including recent floods, local government capacity, socioeconomic conditions and the number of CRS participating communities within a county on CRS participation. They found that more floodplain management activities among counties with recent flood experience, greater hydrological risk and more local jurisdictions within the county also participating in the CRS program led to higher CRS scores. Sadiq and Noonan (2015) examined CRS activities throughout the nation and how they may be affected by flood risk, local government capacity and the socioeconomic attributes of residents, among other factors. They found that more hazard mitigation planning was associated with wealthier, better-educated residents [25]. Similarly, other studies have found that wealthier home owners may invest more in the protection of their property, be less willing to relocate and may be more supportive of local hazard mitigation efforts [44]. In a recent study of Florida counties’ CRS scores, Brody and colleagues found that higher scores were associated with higher socioeconomic capital, recent flood experience and less land area located in a flood plain. Previous research also suggests that the greater amount of floodplain in a county may deter local CRS flood mitigation efforts; the costs of implementing mitigation measures may not outweigh the discount in insurance premiums [24].

These studies suggest that socioeconomic attributes of residents, county government capacity, physical factors, such as elevation, and experience with recent flood events may influence the level of CRS planning. Thus, we include indicators of these conditions and attributes of the parishes (counties) within the south Louisiana study area.

## 2. Materials and Methods

As stated previously, this analysis examines the extent to which southern Louisiana parishes have taken steps to exceed NFIP requirements to reduce local flood risks and identifies the factors that account for variation in the CRS scores among the parishes.

### 2.1. Sample Selection

The sample selected consists of the 35 parishes of South Louisiana, listed below in Table 3. Of those 35 parishes, 15 are in the CRS program. All parishes have been in the CRS program for at least 19 years, except for Lafayette Parish, which joined the program in 2011. We selected only the parishes involved in the CRS program (leaving out smaller municipalities) in order to use readily-available demographic and flood-related data. The only Louisiana parish listed in the CRS program outside of our study area was Caddo Parish in Northwest Louisiana.

### 2.2. Dependent Variable

The dependent variable is taken directly from each parish’s CRS score. We used this score (as opposed to the CRS class level) in order to be able to statistically analyze a continuous variable. The scores range from a low of 0 to the highest parish score of 2213.

The independent variables included in this analysis are summarized below in Table 4. Drawing from recent related research, we chose to include measures of socioeconomic conditions, government capacity and flood exposure risk. Given the relatively small number of parishes in the study area (36), we limited the number of independent variables to be considered in the analysis. The average housing value is included to capture the relative affluence within the county and the value of the properties at risk of flooding. We also included the college education rate among the residents as an indicator of the socioeconomic attributes of the parish. Furthermore, the parish government revenue is included to indicate the public resources available to the county decision makers. The number of municipalities that participate in the CRS program within each parish is included to help indicate the capacity of the parish to development and implement the CRS measures. The presence of more participating jurisdictions may create a stronger base of public support for more proactive, adaptive planning to reduce flood risks. Flood risk is indicated by two variables. First, we included the number of floods over the last five years to indicate exposure to risks and the flood experiences, in addition to possible risk perceptions of the residents. The second measure of flood risk is the mean elevation of the parish, with lower elevation indicating greater flood exposure risk. The independent variables and their data sources are summarized below in Table 4.

### 2.3. Data Analysis

We began by conducting a Pearson correlation analysis among the variables to identify any potentially highly correlated independent variables. Next, we conducted a multiple regression analysis to determine the relative statistical associations between the independent variables and the CRS scores. We conducted the analysis in SPSS Version 21. The choice of the analysis was appropriate given that the dependent variable, the CRS score, is a continuous variable. The descriptive statistics for the dependent variable and the independent variables included in the study are shown in Table 5.

## 3. Results and Discussion

**Figure 1:**
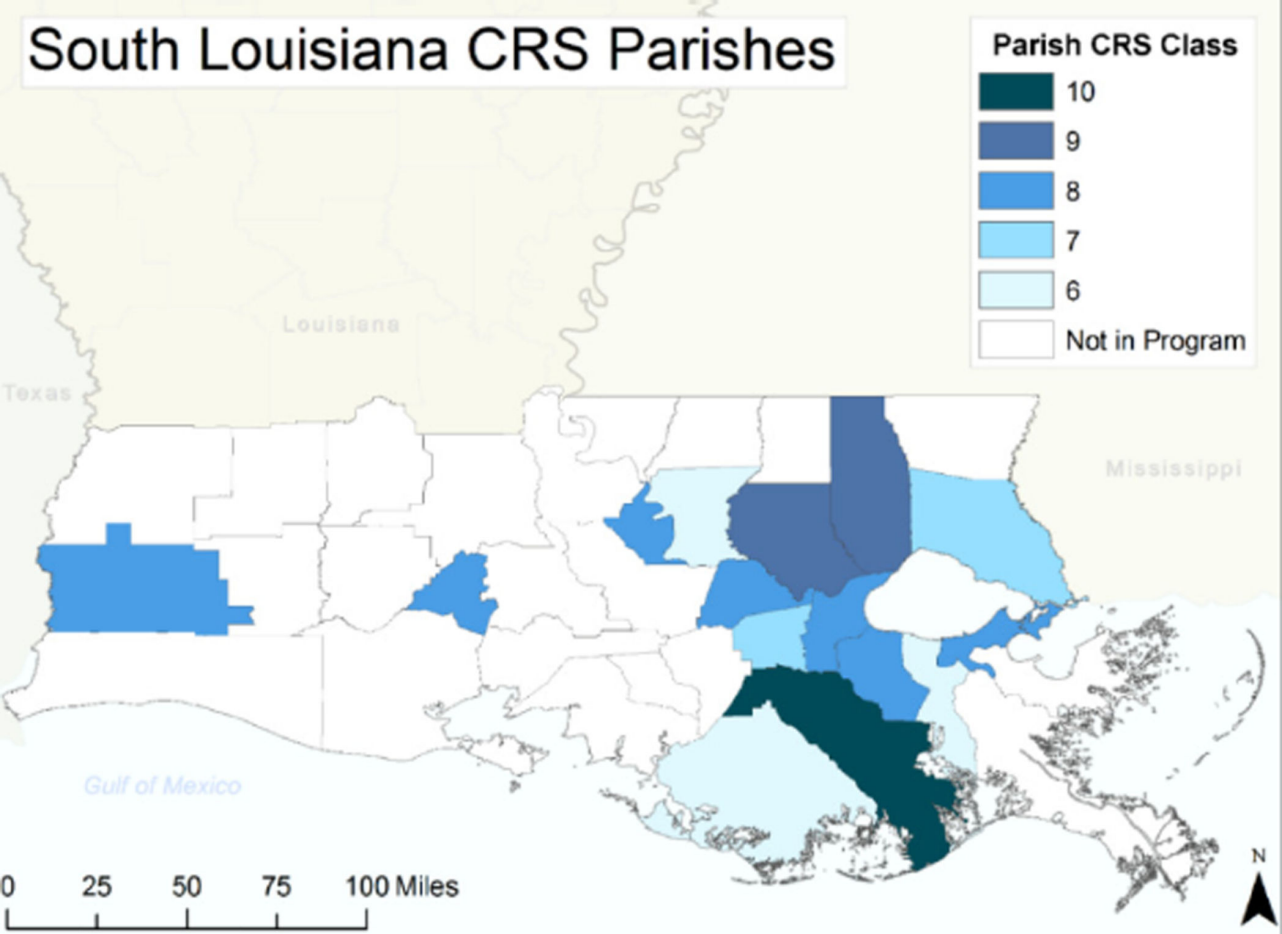
Map of the study area with CRS participating counties coded by CRS class ranking.

Our first research objective is to examine the level of participation in the CRS program among counties (parishes) in south Louisiana. Of the 35 parishes in the study area, 15 have achieved CRS class rankings. Figure 1 illustrates the location and CRS class of these jurisdictions. The majority of the participating parishes are located in the southeast portion of Louisiana, along with Lafayette in the central region and Calcasieu on the west side of the study area. Lafourche parish is rated a Class 10, which means it was once in the program, but is now inactive.

The second research objective is to identify key factors that may explain variation in the CRS scores. We conducted a Pearson bi-variate correlation analysis to identify statistically-significant associations between the variables in the analysis before constructing the multiple regression model. We considered a significant correlation value of 0.7 or higher to indicate a high degree of multicollinearity. We found that the percentage of college-educated residents was significantly and positively associated with the average housing value within the parishes, with a Pearson R of 0.770. Therefore, we did not include both variables in the regression analysis. We selected the housing value variable for further analysis, because it provides an indicator of not only economic resources, but also tangible assets that may be damaged by floods. Since none of the other independent variables were found to have Pearson R values of greater than 0.7, these five were retained for inclusion in the regression analysis. The results of the Pearson analysis are summarized below in Table 6.

Next, we conducted a multiple regression analysis using the five selected independent variables. The results of the analysis are summarized in Table 7 below.

**Figure 2:**
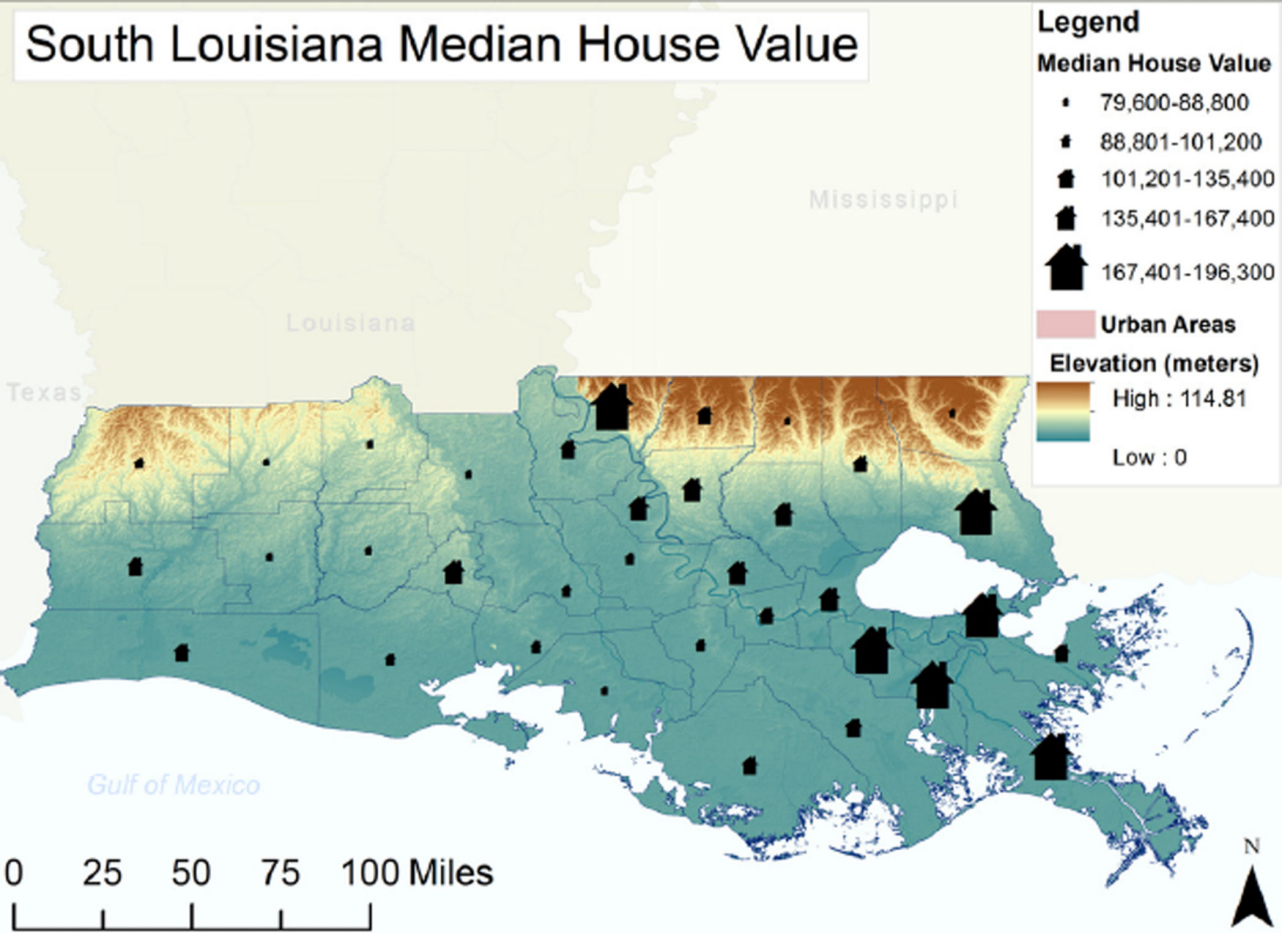
Map of housing values and elevations among parishes.

The regression analysis yielded an adjusted R squared of 0.571, indicating that these five independent variables explained 57% of the variation in the parish CRS scores. Higher CRS scores are associated most closely with higher average housing values. The distribution of housing values within the study area is illustrated in Figure 2. This is not a surprising finding and indicates that parishes with more valuable built assets and likely more affluent residents have implemented more of the suggested actions to reduce flood risks. This finding is consistent with those of Sadiq and Noonan (2015) in their study of a sample of CRS communities throughout the nation and also those of Brody and colleagues (2009) in their examination of Florida counties [25,46]. The finding also is in keeping with prior research examining the more general attributes of communities that appear to enhance overall resilience to a range of large-scale disturbances. The findings of Lam *et al.*, 2015, and Cutter *et al.*, 2009 [37,47], for example, consistently point to the importance of socioeconomic resources in building resilience.

Regarding the capacity of the county governments, the presence of more municipalities participating in the CRS program within a parish is significantly associated with higher CRS scores at the parish level. This finding is not surprising and supports the conclusions of Landry and Li in their study of North Carolina counties’ CRS participation from 1991 to 1996 [45]. The presence of more “nested municipalities” that are involved in the hazard mitigation planning encouraged by the CRS within a county may well increase the level of public awareness of the benefits of the collective actions and may provide a larger base of expertise and technical information to support the formulation and implementation of these measures. These factors may be particularly helpful to parish decision makers in Louisiana given the state’s lack of a well-established culture of land use planning. The revenue base of the county government was not found to be significantly associated with the level of CRS program implementation.

The finding that average county elevation is not significantly associated with CRS planning activities is consistent with previous research of Florida counties conducted by Zahran in 2010 [24]. In Louisiana, it appears that mere location in a more low-lying and presumably more flood-prone area is not sufficient to prompt planners and policy makers to formulate and implement more CRS measures. One reason could be that planning and floodplain management activities in lower lying counties may be more difficult and expensive due to the larger amount of floodplain area [24]. We were somewhat surprised that the number of past flood events was not found to be related to CRS scores. This finding differs from the Zahran study in 2010 and suggests that among the south Louisiana parish decision makers, flood experience may be not sufficient to encourage the type of collective action specified by the CRS program. This finding may be further evidence of the short-term nature of risk perceptions found in earlier studies; that is, that past floods may fade from the memory of both residents and policy makers rather quickly [18]. The interest surrounding the development of collective plans and strategies for flood protection may not be as urgent a public policy issue as time progresses, if floods are not experienced regularly.

## 4. Conclusions

The objective of the analysis was to examine the context under which coastal parishes (counties) may be more likely to take steps to make themselves safer through floodplain management and other measures encouraged by the CRS program. The results of the regression analysis indicate that higher CRS scores are found in parishes with higher housing values and with a higher number of municipalities within the parish that also participate in the CRS program. Surprisingly, indicators of greater exposure to flood risks, including lower mean elevation and past flood events, were not found to be significantly associated with greater participation.

The CRS program is an important effort by the federal government to encourage local governments to become more proactive and adaptive in flood hazard mitigation planning. As the National Flood Insurance Program (NFIP) faces major deficits, this incentive-based approach to spur more collective floodplain management activities among county and local jurisdictions is compelling. Are there key contextual factors that may affect the extent to which local jurisdictions are willing or able to participate?

This analysis of southern Louisiana parishes indicates that acceptance of the incentives offered through the CRS program to move toward more collective hazard mitigation efforts may be influenced to a large extent by the socioeconomic attributes of the parish. These findings are consistent with prior related research in suggesting that more affluent communities with more valuable housing and property are more likely to achieve higher CRS scores. Furthermore, consistent with the Landry and Li study of counties in North Carolina, this analysis found that the presence of more local jurisdictions within the parish that also are participating in the CRS program is associated with higher county CRS scores [45]. The presence of these local CRS programs within the south Louisiana parishes may introduce more public support for the planning measures along with more technical expertise and resources for their implementation. By contrast, parishes with fewer socioeconomic resources and parish government capacity may face additional obstacles to formulating and implementing measures for collective flood hazard mitigation. This may be especially relevant in states like Louisiana, without a well-established history of local planning and where flood risks and NFIP premiums can only be expected to increase. As a result, the CRS program administrators may need to include additional outreach and technical assistance to lower income jurisdictions to encourage more collective action to reduce flood exposure risks to residents of flood-prone communities.

## Figures and Tables

**Table 1:** The Community Rating System (CRS) activities and credit point system [6,7].

**Series 300**	**Public Information**	**Maximum Points**	**National Average**	**Louisiana Average**

310	Elevation Certificates	162	68	66
320	Map Information Service	140	140	140
330	Outreach Projects	380	99	80
340	Hazard Disclosure	81	14	15
350	Flood Protection Information	102	45	46
360	Flood Protection Assistance	71	47	51
370	Flood Insurance Promotion *	0	0	0
	Total	936	413	398

**Series 400**	**Mapping and regulations**	**Maximum Points**	**National Average**	**Louisiana Average**

410	Additional Flood Data	1346	89	56
420	Open Space Preservation	900	182	93
430	Higher Regulatory Standards	2740	291	167
440	Flood Data Maintenance	239	97	82
450	Stormwater Management	670	111	71
	Total	5895	770	469

**Series 500**	**Flood Damage Reduction**	**Maximum Points**	**National Average**	**Louisiana Average**

510	Floodplain Management Planning	359	129	135
520	Acquisition and Relocation	3200	237	121
530	Flood Protection	2800	79	68
540	Drainage System Maintenance	330	201	224
	Total	6689	646	548

**Series 600**	**Flood Preparedness**	**Maximum Points**	**National Average**	**Louisiana Average**

610	Flood Warning Program	255	93	110
620	Levee Safety	900	93	0
630	Dam Safety	175	63	69
	Total	1330	249	179

Note:

* Flood Insurance Promotion, Activity 370, was a new activity in 2013, and therefore, no community has earned these points as of publication. Below is a summary of each activity, taken directly from the 2014 CRS Manual.

**Table 2:** The CRS points and classification system [6]. SFHA, Special Flood Hazard Area.

Credit Points (Score)	Class	Premium Reduction		Number of Louisiana Parishes

		(SFHA)	Non-SFHA	
4500+	1	45%	10%	0
4000–4499	2	40%	10%	0
3500–3999	3	35%	10%	0
3000–3499	4	30%	10%	0
2500–2999	5	25%	10%	0
2000–2499	6	20%	10%	3
1500–1999	7	15%	5%	2
1000–1499	8	10%	5%	8
500–999	9	5%	5%	2
0–499	10	0%	0%	1

**Table 3:** Parishes and CRS points in the study area.

Parish	CRS Points	Parish	CRS Points
Acadia	-	Plaquemines	-
Allen	-	Pointe Coupee	-
Ascension	1690	St. Bernard	-
Assumption	-	St. Charles Parish	1730
Beauregard	-	St Helena	-
Calcasieu	1392	St. James Parish	1547
Cameron	-	St. John the Baptist	1006
East Baton Rouge	2068	St. Landry	-
East Feliciana	-	St Martin	-
Evangeline	-	St. Mary	-
Iberia	-	St. Tammany	1716
Iberville	-	Tangipahoa	642
Jefferson	2213	Terrebonne	2021
Jefferson Davis	-	Vermilion	-
Lafayette	1329	Washington	-
Lafourche	0	West Baton Rouge	1638
Livingston	845	West Feliciana	-
Orleans	1039		

**Table 4:** Independent variables.

Variable	Variable Operation	Data Source
Socioeconomics		
Median Home Value College-Education Rate	Value is an estimate of how much the property (house and lot) would sell for if it were for sale. Includes only specified owner-occupied housing units. Dollars expressed in $10,000 increments. The percentage of residents with college degrees	U.S. Census Bureau, 2010
Government Capacity		
2010 Government Revenue	Total expenditures for the parish government for the year 2010.	Parish Assessors’ Offices
Number of CRS Communities	Number of participating CRS communities located in each participating CRS parish	NFIP
Exposure		
Average Elevation	The number of meters above base sea level	United States Geological Survey Coastal National Elevation Database Project-Topobathymetric Digital Elevation Model: (USGS CoNED TBDEM) 3 m, 2014
Number of total flood events	The total number of flood events, 2006–2010	Spatial Hazards Events and Losses Database for the United States (SHELDUS), 2006–2010

**Table 5:** Independent variables’ descriptive statistics.

Independent Variable	N	Minimum	Maximum	Mean	Std. Deviation
Total CRS Points 2013 (score)	35	0	2213	596.46	797.02

Socioeconomics					

Median Home Value	35	79,600.00	196,300.00	125,914.28	37,473.83
College Educated Rates	35	9.7	34.2	16.63	6.58

Government Capacity					

2010 Government Revenue per Person	35	6.71	157.94	28.75	27.38
CRS communities	35	0.00	4.00	0.77	1.28

Exposure					

Average Elevation	35	−0.73	61.77	12.69	18.55
Number of total Flood Events 2006–2010	35	0	21	2.40	3.86

Valid N (listwise)	35				

**Table 6:** Pearson correlation analysis.

Independent Variable	Total CRS Points 2013	# CRS Communities per Parish	Average Elevation	Median House Value per 1k	College- Educated Rate	# of Total Flood Events 2006–2010	2010 Government Revenue per Person
Total CRS Points 2013 (score)	1.000	0.65 ***	−0.28 *	0.66 ***	0.69 ***	0.11	−0.28

**Socioeconomics**							

Median Home Value	0.66 ***	0.49 ***	−0.22	1.00	0.77 ***	−0.12	0.01
College Educated Rate	0.69 ***	0.64 ***	−0.14	0.77 ***	1.00	0.13	−0.15

**Government Capacity**							

CRS Communities	0.65 ***	1.00	−0.15	0.49 ***	0.64 ***	0.17	−0.27
2010 Government Revenue per Person	−0.28	−0.27	−0.09	0.009	−0.15	−0.23	1.00

**Exposure**							

Average Elevation	−0.28 *	−0.15	1.00	−0.22	−0.14	0.010	−0.09
# of total Flood Events 2006–2010	0.11	0.17	0.010	−0.12	0.13	1.00	−0.23

Notes: *N* = 35;

*** *p* < 0.01;

** *p* < 0.025;

* *p* < 0.05.

**Table 7:** Multiple regression analysis results.

Model	Unstandardized Coefficients		Standardized Coefficients	t	Significance

	B	Standard Error	Beta		
(Constant)	−594.70	386.90		−1.54	0.13

Socioeconomics					

Median Home Value	0.01	0.00	0.46	3.40	0.00
Government Capacity CRS Communities	218.80	85.62	0.35	2.56	0.02
2010 Government Revenue per Person	−5.38	3.51	−0.18	−1.53	0.14

Exposure					

Average Elevation	−6.58	4.98	−0.15	−1.32	0.20
# of total Flood Events 2006–2010	13.53	24.39	0.07	0.55	0.58

Notes: Dependent variable: total CRS points 2013. Model *p* < 0.001, adjusted R squared = 0.571, *N* = 35.
